# Integrative Transcriptomic and Metabolic Analyses Provide Insights into the Role of Trichomes in Tea Plant (*Camellia Sinensis*)

**DOI:** 10.3390/biom10020311

**Published:** 2020-02-16

**Authors:** Hongli Cao, Jiamin Li, Yijun Ye, Hongzheng Lin, Zhilong Hao, Naixing Ye, Chuan Yue

**Affiliations:** College of Horticulture, Fujian Agriculture and Forestry University/Key Laboratory of Tea Science in Universities of Fujian Province, Fuzhou 350002, China; lili9885@126.com (H.C.); aefldfr@163.com (J.L.); youbingpuzi@163.com (Y.Y.); linhongzheng2010@126.com (H.L.); haozhilong@126.com (Z.H.); ynxtea@126.com (N.Y.)

**Keywords:** tea plant, trichomes, trichomes development, secondary metabolites, volatile aroma

## Abstract

Trichomes, which develop from epidermal cells, are regarded as one of the key features that are involved in the evaluation of tea quality and tea germplasm resources. The metabolites from trichomes have been well characterized in tea products. However, little is known regarding the metabolites in fresh tea trichomes and the molecular differences in trichomes and tea leaves per se. In this study, we developed a method to collect trichomes from tea plant tender shoots, and their main secondary metabolites, including catechins, caffeine, amino acids, and aroma compounds, were determined. We found that the majority of these compounds were significantly less abundant in trichomes than in tea leaves. RNA-Seq was used to investigate the differences in the molecular regulatory mechanism between trichomes and leaves to gain further insight into the differences in trichomes and tea leaves. In total, 52.96 Gb of clean data were generated, and 6560 differentially expressed genes (DEGs), including 4471 upregulated and 2089 downregulated genes, were identified in the trichomes vs. leaves comparison. Notably, the structural genes of the major metabolite biosynthesis pathways, transcription factors, and other key DEGs were identified and comparatively analyzed between trichomes and leaves, while trichome-specific genes were also identified. Our results provide new insights into the differences between tea trichomes and leaves at the metabolic and transcriptomic levels, and open up new doors to further recognize and re-evaluate the role of trichomes in tea quality formation and tea plant growth and development.

## 1. Introduction

Trichomes are developed from epidermal cells and they can be uni- or multicellular, glandular or non-glandular, branched or unbranched, and extensively distributed on plant tissues, playing critical roles in plant growth and the development and stress responses [1,2,3,4,5,6]. In tea plants (*Camellia sinensis*), trichomes are one of the main characteristics that are critical for tea plant breeding and quality formation of tea products. Trichomes are mainly present on the abaxial surface and stems of tender leaves and abscise as the shoots mature [7]; therefore, teas with an abundance of trichomes are usually processed as tender and fresh tea shoots, and an abundance of trichomes seems to be a key feature indicating high quality. Previously, we observed that tea plants primarily have a single type of unicellular, unbranched, and non-glandular trichome [7]. It has been well established that the most notable difference between glandular and non-glandular trichomes is that glandular trichomes have the capability to synthesize, store, and secrete a wide variety of metabolites, including polysaccharides, organic acids, proteins, terpenoids, alkaloids, and polyphenols [1,5,8,9], suggesting that tea trichomes may not have the capability to synthesize, store, or secrete these metabolites. Nonetheless, several previous studies showed that tea trichomes also had a wide variety of metabolites, such as catechins, caffeine, amino acids, and aromatic compounds. Given that almost all tea plant cultivars or germplasm resources have trichomes on the tender shoot surfaces, if these trichomes do not synthesize metabolites, what is their function in the tea plant?

Trichomes have been regarded as a perfect system in which to study cell development and secondary metabolite synthesis in the model plants *Arabidopsis* [10,11,12,13,14,15,16], tomato [17,18,19,20,21,22,23], and *Artemisia annua* [24,25,26,27,28,29,30], and many genes that are involved in trichome differentiation have been identified, especially various transcription factors (TFs) encoding genes, such as positive regulators of *GLABROUS 3* (*GL1*, *GL2*, and *GL3*) and *ENHANCER OF GLABROUS 3* (*EGL3*), *TRANSPARENT TESTA 1* (*TTG1* and *TTG2*) and repressors of *TRIPTYCHON* (*TRY*), *CAPRICE* (*CPC*), *ENHANCER OF TRY*, *CPC 1* (*ETC1*, *ETC2,* and *ETC3*), and *TRICHOMELESS 1* (*TCL1* and *TCL2*) [6,12,13,16]. Moreover, an increasing number of studies have indicated that trichomes seem to be an excellent model in which to study the molecular mechanisms of secondary cell wall deposition and fiber synthesis [31,32], which have been well studied in plants. Recently, we performed RNA-Seq analysis while using hairy and hairless cultivars, and many genes were identified to investigate the genes involved in tea trichome formation [7]. Studies on fresh trichomes, especially comparisons of trichomes and tea leaves *per se* at the molecular level, are lacking since it is difficult to collect fresh trichomes from tea shoots and all trichomes used in previous studies were isolated from tea products that were subjected to a series of processes.

In the present study, we first developed a method to collect trichomes from fresh tender shoots, which could satisfy the research requirements at the molecular level. The major non-volatile metabolites, including catechins, caffeine, amino acids, and aromatic compounds, in the trichomes were determined while using ultra-high performance liquid chromatography coupled with triple quadrupole mass spectrometry (UPLC-QqQ-MS) and gas chromatography mass spectrometry (GC-MS). Most importantly, the trichome and tea leaf samples were subjected to RNA-Seq analysis, and the differentially expressed genes (DEGs) were identified and compared. When combined with the metabolite contents and gene expression patterns, we showed that tea trichomes might not have obvious contributions, as previously expected, to tea biochemical quality, but might only contribute to tea appearance quality as well as function as the first barriers against a variety of abiotic and biotic stresses.

## 2. Materials and Methods

### 2.1. Plant Materials and Trichomes Collection

In this study, the hairy tea plant *Camellia sinensis* var. Fudingdabaicha growing in a natural tea garden at Fujian Agriculture and Forestry University (Fuzhou, China, 26°05′ N, 119°18′ E) was used as the plant material. One-bud-and-one-leaf tissue samples were collected and then frozen quickly using liquid nitrogen, and trichome isolation was performed by shaking the tissues in liquid nitrogen, as previously described by Spyropoulou, et al. [33], with minor modifications, to isolate fresh trichomes from tender tea shoots in April 2019. Briefly, the tender tissues were collected and stored in a 50 mL tube and quickly frozen in liquid nitrogen. After that, the tube was held on a motor to shake the samples for 10 sec. until the trichomes fell off, and the trichomes were collected from the tubes using spoons. To isolate enough trichomes for further analysis, at least 400 shoots of one-bud-and-one-leaf were sampled from over 40 tea individuals for each replicate, and three biological replicates were used to investigate. The isolated trichomes and processed leaves were used for total RNA extraction and metabolite detection.

### 2.2. Determination of Non-Volatile and Volatile Compounds in Tea Trichomes and Leaves

Tissues of trichomes and processed leaves were dried by freezing and ground to fine powders while using mortars and pestles. 30 mg of each sample was extracted using 1.2 mL of 70% (*v*/*v*) methanol and then vortexed and sonicated at 25 °C for 20 min. to extract the main non-volatile metabolites in the tissues. After centrifugation (10 min., 12,000×), the supernatants were transferred and filtered through a 0.22 μm PVDF filter (Millipore) and stored at −20 °C until analysis. The contents of amino acids, catechins and caffeine were determined while using UPLC-QqQ MS-based targeted quantifications based on authentic standard, as previously described by Chen, et al. [34].

The volatile compounds of fresh trichomes and processed leaves were extracted and determined, as previously described by Dong, et al. [35] and Zeng, et al. [36], with minor adjustments. Briefly, 250 mg of each finely powdered fresh sample was extracted while using dichloromethane (2 mL), and ethyl *n*-decanoate (5 nmol) was added as an internal standard. After overnight shaking, the extraction solution was collected, dried using anhydrous sodium sulfate, and concentrated to 100–200 μL under a stream of nitrogen. The extract (1 μL) was then subjected to GC-MS that was carried out on a GC-MS QP2010 SE (Shimadzu Corporation, Kyoto, Japan) that was equipped with GC-MS Solution software (Version 2.72, Shimadzu Corporation, Japan). Samples were injected into the GC injection port and held at 230 °C for 1 min., with all injections made in splitless mode. The aroma compounds were separated on a SUPELCOWAX 10 column (30 m × 0.25 mm × 0.25 μm, Supelco Inc., Bellefonte, PA, USA). Helium was used as the carrier gas, with a flow rate of 1 mL/min. The initial GC oven temperature was 60 °C for 3 min., which was ramped up to 240 °C at a rate of 4 °C/min, and then held at 240 °C for 20 min. The mass spectrometer was operated in full scan mode (mass range, *m*/*z* 40–200). Some volatile compounds were identified by direct comparison with authentic standards and then quantified according to their calibration curves. Some aromatic compounds without authentic standards were tentatively identified by comparison with mass spectra, and the relative content of each compound was calculated by comparison with the peak area of the internal standard. Three biological replicates were used for each tissue.

### 2.3. RNA Isolation, Library Preparation, and Illumina Sequencing

Total RNA was extracted from tea leaves and trichomes using the TIANGEN RNAprep Pure kit (TIANGEN, Beijing, China), and genomic DNA elimination by treated with DNase I (Invitrogen, Carlsbad, CA, USA) and RNA quality and quantity determination were performed, as previously described by Yue, et al. [7]. Only RNA samples with an A260/A280 > 1.8, A260/A230 > 1.8, and RNA integrity number (RIN) > 8 were used for library construction. A total amount of 3 μg of RNA per sample was used to generate sequencing libraries while using the NEBNext^®^ UltraTM RNA Library Prep Kit for Illumina^®^ (NEB, USA), following the manufacturer’s recommendations. To preferentially select cDNA fragments 250~300 bp in length, the library fragments were purified with the AMPure XP system (Beckman Coulter, Beverly, USA). Subsequently, 3 μL of USER Enzyme (NEB, USA) was used with size-selected, adaptor-ligated cDNA at 37 °C for 15 min. followed by 5 min. at 95 °C before PCR. Afterwards, the PCR products were purified (AMPure XP system), and the library quality was assessed on the Agilent Bioanalyzer 2100 system.

Clustering of the index-coded samples was performed on a cBot Cluster Generation System using TruSeq PE Cluster Kit v3-cBot-HS (Illumina), according to the manufacturer’s instructions. After cluster generation, the library preparations were sequenced on an Illumina HiSeq platform (Novogene, Beijing, China).

### 2.4. Transcriptome Data Processing

The quality of raw data was processed through in-house Perl scripts. Clean reads were obtained by removing reads containing adaptors, reads containing poly-N and low-quality reads from raw data. The Q20, Q30, and GC contents of the clean data were calculated. All the downstream analyses were based on clean data with high quality.

The paired-end clean reads of each sample were aligned to the tea plant genome while using Hisat2 v2.0.5 with the default parameters [37]. The feature Counts v1.5.0-p3 was used to count the read numbers mapped to each gene [38]. Subsequently, the fragments per kilobase of transcript per million mapped reads (FPKM) value of each gene was calculated based on the length of the gene and read count mapped to that gene. The DESeq2 R package (1.16.1) was used to identify DEGs [39]. The resulting P-values were adjusted while using Benjamini and Hochberg’s approach for controlling the false discovery rate, and the unigenes with a |log_2_ Fold Change| > 1 and adjusted *p*-value < 0.05 were considered to be differentially expressed.

The DEGs were subjected to Gene Ontology (GO) enrichment analysis while using the clusterProfiler R package, and the GO terms with corrected *p*-values < 0.05 were considered significantly enriched [40]. The clusterProfiler R package was used to test the statistically significant enrichment of the DEGs in Kyoto Encyclopedia of Genes and Genomes (KEGG) pathways to understand the high-level biological functions and pathways of DEGs [41]. An FDR ≤ 0.05 was set as a threshold value to identify enrichment pathways.

### 2.5. Quantitative Real-Time PCR Validation

In total, 21 DEGs were selected for real-time qPCR analysis to validate the transcriptome data. qRT-PCR was carried out while using SYBR Premix Ex Taq™ II (TaKaRa, Dalian, China) and run on a CFX96 Touch Real-Time PCR System (BIO-RAD, California, CA, USA), according to the manufacturer’s protocol. The *Camellia sinensis GADPH* reference gene was used as an internal control, as described in previous studies [7]. Table S1 shows the primers for RT-qPCR. Each sample was repeated in triplicate, and the relative gene expression levels were determined while using the 2^−ΔΔCT^ method. The results are presented as the mean ± standard deviation (SD).

### 2.6. Statistical Analysis

Statistical analyses were performed while using SPSS (SPSS Inc., Chicago, IL, USA) and Prism7 (GraphPad Software Inc., La Jolla, CA, USA). The differences among the means were analyzed using the two-tailed Student’s t test at a 0.01 probability level.

## 3. Results

### 3.1. Comparison of the Key Secondary Metabolites Between Tea Trichomes and Leaves

Nineteen amino acids were determined from tea trichomes and leaves while using UPLC-QqQ-MS. However, threonine was not detected in trichomes, and serine was not detected in leaves. The total contents of amino acids in the leaves was more than 10 times higher than that in the trichomes (Table 1).

The most abundant amino acids in both the trichomes and leaves were theanine, glutamic acid, glutamine, aspartic acid, and arginine, which accounted for 93.1% and 94.4% of the amino acids, respectively. Theanine was the most abundant amino acid in both the leaves and trichomes, containing 4.49 mg/g and 0.76 mg/g of this amino acid, respectively.

The catechin detection results showed that the content of catechins was significantly higher (more than 61 times) in the leaves than in trichomes. Epigallocatechin gallate (EGCG), epicatechin gallate (ECG), epigallocatechin (EGC), and epicatechin (EC) were the most abundant catechins. In leaves, the contents of EGCG, ECG, EGC, and EC were 82.7 mg/g, 28.8 mg/g, 17.7 mg/g, and 4.2 mg/g, respectively, whereas they were 1.1 mg/g, 0.54 mg/g, 0.085 mg/g, and 0.037 mg/g in trichomes (Table 2).

The rutin content in tea leaves (0.39 mg/g) was more than six-fold higher than that in trichomes (0.058 mg/g). The caffeine content in tea leaves (31.2 mg/g) was more than 30-fold higher than that in trichomes (1.01 mg/g).

### 3.2. Comparison of Aromatic Compounds Between Tea Trichomes and Leaves

34 of the main aromatic compounds were identified and quantified while using GC-MS to investigate the aromatic compounds in fresh trichomes and processed tea leaves. Among the tested compounds, the content of phytol was the highest in both trichomes and leaves, followed by 3,5-dimethylbenzaldehyde, 2-phenylethanol, benzyl alcohol, 2-hexenal, (*Z*)-3-hexenol, and linalool, as shown in Table 3.

However, the contents of the majority of aromatic compounds were significantly higher in the tea leaves than in trichomes, especially that of nerol, methyl salicylate, trans-linalool oxide (furanoid), 2-hexenal, trans-2-hexenyl butyrate, linalool, alpha-farnesene, phenylethyl alcohol, (*Z*)-3-hexenol, trans-nerolidol, and linalool oxide (pyranoid), which are recognized as the key fragrant contributors to tea aromatic quality. On the other hand, the contents of (*E*)-2-hexenol, hexyl butanoate, and trans-2-hexenyl hexanoate were significantly accumulated in trichomes, and cyclohexanone, cis-3-hexenyl butyrate, 2-ethylhexanol, and benzeneacetaldehyde also had high, but not significantly higher, contents in trichomes.

### 3.3. RNA Sequencing, Reference Genome Alignment, and New Gene Annotation

Six cDNA libraries were constructed while using trichomes and leaves to investigate the differences in genes between tea trichomes and leaves (each sample was repeated three times). In total, 353.0 million high-quality clean reads were generated from six libraries, and the sequence data were deposited in NCBI (SRA accession: PRJNA560722). The average clean bases, Q20 values, and Q30 values of each sample were 8.83 Gb, 97.88%, and 93.95%, respectively (Table S2). The read mapping ratio per sample to the reference genome was 86.55–90.50% (Table S2). A total of 11460 transcripts were identified as novel genes and annotated to the Pfam, SUPERFAMILY, GO, and KEGG databases.

### 3.4. Differential Expression Profiling between Tea Trichomes and Leaves

A volcano plot was constructed to determine the genes that were significantly changed between trichomes and leaves. In total, 6560 DEGs were identified from the comparison of trichomes and leaves according to the parameters (*p*-value < 0.01 and |log_2_ Fold Change|>1). Among these DEGs, there were more upregulated DEGs (4471) than downregulated DEGs (2089), while the fold change values of the upregulated DEGs were also larger than that of downregulated DEGs (Figure S1).

**Figure 1:**
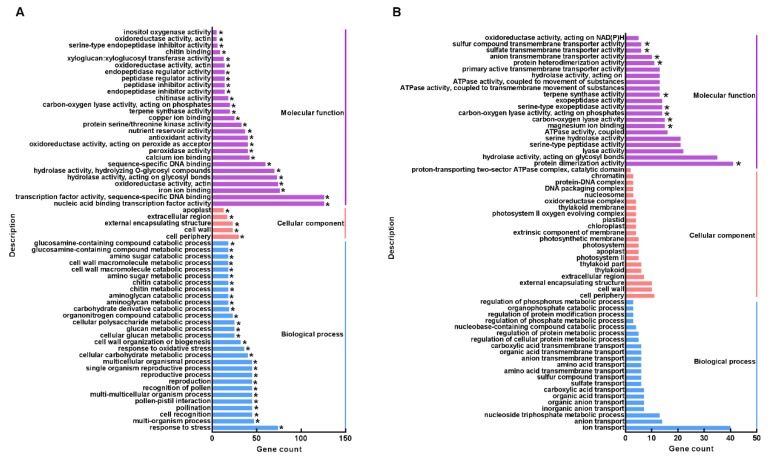
Gene ontology term enrichment analysis of the up- (**A**) and downregulated (**B**) differentially expressed genes (DEGs) in the comparison of trichomes vs. leaves. * indicates the significantly enriched terms.

GO term and KEGG pathway enrichment analyses were performed on the upregulated and downregulated DEGs, respectively, to analyze the functional enrichment of the DEGs in trichomes in comparison with leaves. For upregulated DEGs, 62 GO terms, including 29 biological process (BP) terms, 27 molecular function (MF) terms, and five cellular component (CC) terms, were significantly enriched (*p ≤* 0.05). Among these terms, the most enriched components were categorized as nucleic acid binding transcription factor activity (GO:0001071), transcription factor activity, sequence-specific DNA binding (GO:0003700), iron ion binding (GO:0005506), oxidoreductase activity, acting on paired donors, with the incorporation or reduction in molecular oxygen (GO:0016705), response to stress (GO:0006950), hydrolase activity, acting on glycosyl bonds (GO:0016798), hydrolase activity, hydrolyzing O-glycosyl compounds (GO:0004553), sequence-specific DNA binding (GO:0043565), and multiorganism process (GO:0051704) (Figure 1A).

**Figure 2:**
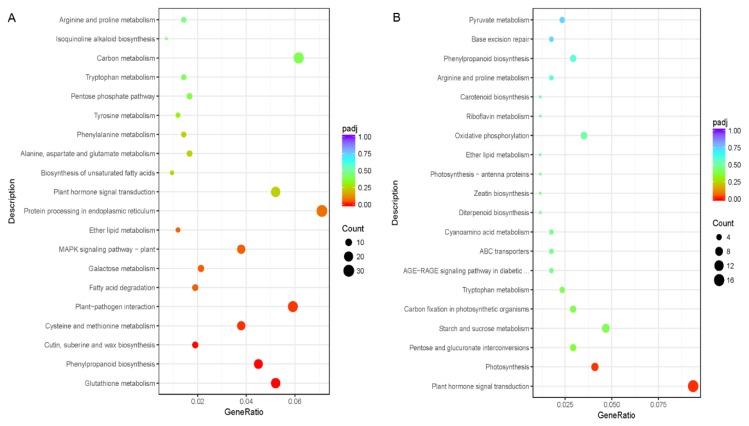
Kyoto Encyclopedia of Genes and Genomes (KEGG) pathway enrichment analysis of the up- (**A**) and downregulated (**B**) DEGs in the comparison of trichomes vs. leaves. The top 20 statistically significantly enriched KEGG pathways for each treatment are shown.

Additionally, KEGG pathway enrichment analyses showed that these DEGs could be mapped to 96 pathways, and the pathways “phenylpropanoid biosynthesis (ath00940)”, “glutathione metabolism (ath00480)”, “cutin, suberine and wax biosynthesis (ath00073)”, “cysteine and methionine metabolism (ath00270)”, and “plant-pathogen interaction (ath04626)” were significantly enriched (Figure 2A).

The downregulated DEGs were categorized into 749 terms that could be classified into BP, MF, and CC categories, whereas all 10 significantly enriched terms were in the MF category, including carbon-oxygen lyase activity, acting on phosphates (GO:0016838), terpene synthase activity (GO:0010333), carbon-oxygen lyase activity, acting on phosphates (GO:0016838), serine-type exopeptidase activity (GO:0070008), protein dimerization activity (GO:0046983), sulfate transmembrane transporter activity (GO:0015116), sulfur compound transmembrane transporter activity (GO:1901682), anion transmembrane transporter activity (GO:0008509), protein heterodimerization activity (GO:0046982), and magnesium ion binding (GO:0000287) (Figure 1B). On the other hand, KEGG pathway analyses showed that, although 2200 downregulated DEGs were classified into 76 pathways, only the “plant hormone signal transduction enrichment (ath04075)” and “photosynthesis (ath00195)” pathways were significantly enriched (Figure 2B), indicating that the tea trichomes have little or no photosynthetic ability.

### 3.5. Differential Expression Profiling of Genes Involved in the Characteristic Metabolite Biosynthesis Pathways Between Tea Trichomes and Leaves

**Figure 3:**
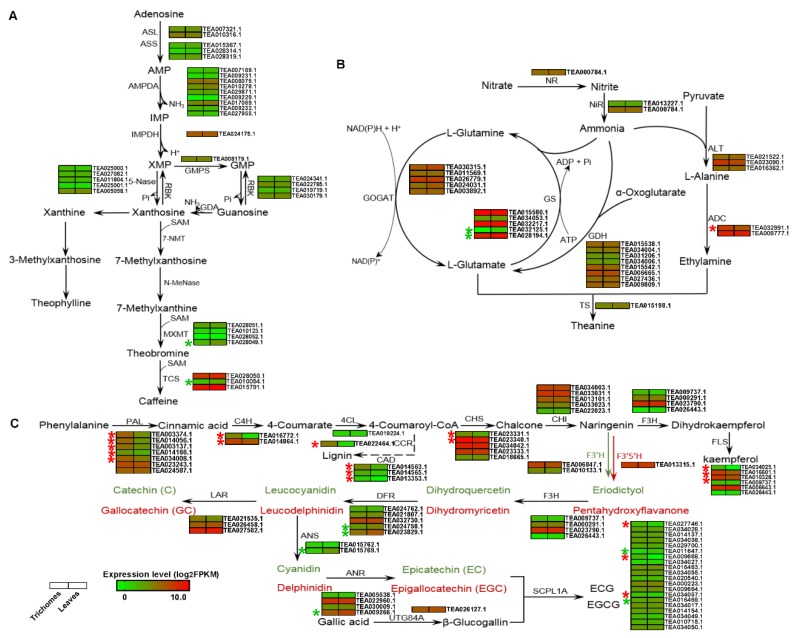
Expression profiles of key genes that are involved in the characteristic metabolite biosynthesis pathways in tea plants. The key genes involved in the caffeine biosynthesis pathway (**A**), theanine biosynthesis pathway (**B**), and catechin biosynthesis pathway (**C**) were identified, and their expression levels in the trichomes and tea leaves are represented by heat maps. The significantly upregulated and downregulated transcripts are shown with red and green asterisks, respectively. ASL, adenylosuccinate lyase; ASS, adenylosuccinate synthetase; AMPD, AMP deaminase; IMPDH, IMP dehydrogenase; GMPS, guanosine monophosphate synthase; RBK, ribokinase; 5′-Nase, 5′-nucleotidase; 7-NMT, 7-methylxanthosine synthase; *N*-MeNase, N-methylnucleotidase; MXMT, theobromine synthase; TCS, tea caffeine synthase; NR, nitrate reductase; NiR, nitrite reductase; GS, glutamine synthetase; GOGAT, glutamate synthase; GDH, glutamate dehydrogenase; ALT, alanine aminotransferase; ADC, argininedecarboxylase; TS, theanine synthetase; PAL, phenylalanine ammonia-lyase; C4H, cinnamic acid 4-hydroxylase; 4CL, 4-coumarate-CoAligase; CCR, cinnamoyl-CoA reductase; CAD, cinnamyl-alcohol dehydrogenase; CHS, chalcone synthase; CHI, chalcone isomerase; F3H, flavanone 3-hydroxylase; F3′H, flavonoid 3′-hydroxylase; F3′,5′H, flavonoid 3′,5′-hydroxylase; FLS, flavonol synthase; DFR, dihydroflavonol 4-reductase; ANS, anthocyanidin synthase; ANR, anthocyanidin reductase; LAR, leucocyanidin reductase; UTG84A, UDP-glycosyltransferase 84A; SCPL1A, serine carboxypeptidase-like 1A. The genes’ fragments per kilobase of transcript per million mapped reads (FPKM) values were listed in the supplementary material Table S3.

The expression patterns of the target genes that are involved in the biosynthetic pathways of theanine, catechins, and caffeine were screened and analyzed. 32 genes that were involved in the caffeine biosynthesis pathway were identified, and the expression profiles showed that 20 genes were downregulated and 12 genes were upregulated in the trichomes, as shown in Figure 3A. However, among these genes, only the expression levels of caffeine synthase (TEA010054) and theobromine synthase (TEA028049) were downregulated at a significant level (log_2_ FoldChange > 1.0). Additionally, 27 genes involved in the theanine biosynthesis pathway were identified (Figure 3B). 

Among these genes, two glutamine synthetase (GS)-encoding genes (TEA032125 and TEA028194) were significantly downregulated (log_2_ FoldChange >1.0), and arginine decarboxylase (ADC, TEA032991) was upregulated in tea trichomes. We found that the theanine synthase gene (TS, TEA015198) was also downregulated, but not at a significant level. Seventy-eight genes, including 41 upregulated and 31 downregulated genes that were involved in regulatory catechin biosynthesis, were isolated from the trichomes vs. leaves dataset (Figure 3C). Among these genes, 14, including phenylalanine ammonia (PAL, TEA003374, TEA014056, TEA003137, TEA014166, and TEA034008), cinnamate 4-hydroxylase (C4H, TEA016772, and TEA014864), chalcone synthase (CHS, TEA023331, and TEA023340), flavonol synthase (FLS, TEA034025, TEA016601, and TEA010328), serine carboxypeptidase 1A (SCPL1A, TEA009668, TEA027746, and TEA034057), were significantly upregulated in trichomes, whereas six genes, including dihydroflavonol 4-reductase (DFR, TEA024758, and TEA023829), anthocyanidin synthase (ANS, TEA015769), anthocyanidin reductase (NAR, TEA009266), and SCPL1A (TEA016469 and TEA011647) were significantly downregulated in trichomes. Interestingly, the transcripts of cinnamoyl-CoA reductase (CCR, TEA014563, TEA014565, and TEA013353) and cinnamyl-alcohol dehydrogenase (CAD, TEA022464), which facilitate the synthesis of lignin while using 4-coumaroyl-CoA, were considerably upregulated in the trichomes.

### 3.6. Differential Expression Profiling of Tfs Between Tea Trichomes and Leaves

**Figure 4:**
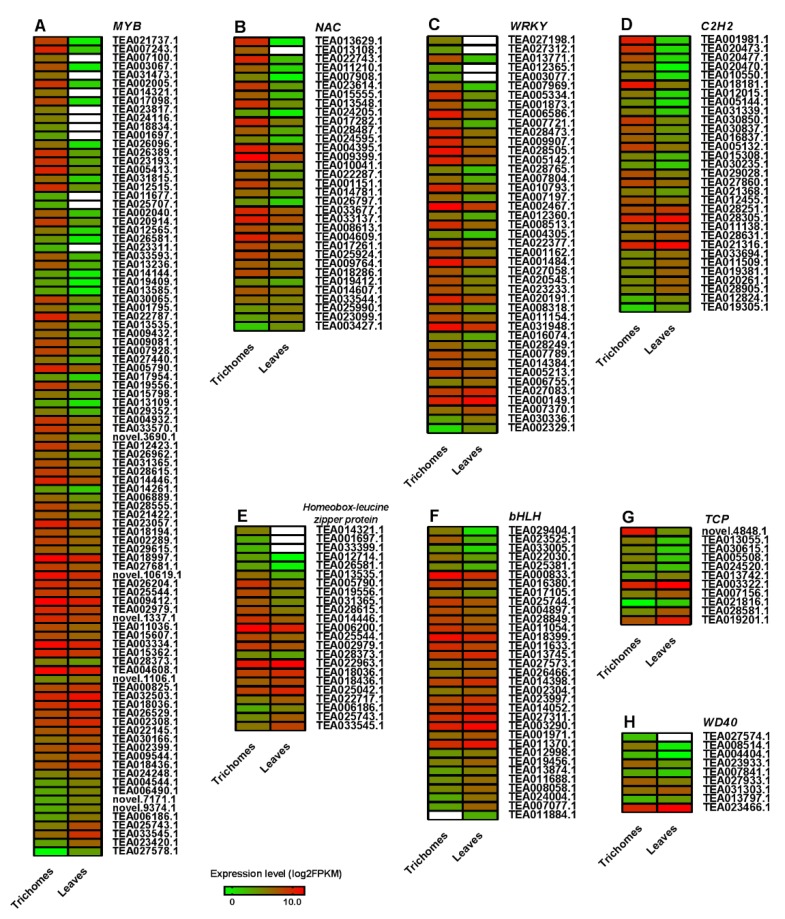
Expression patterns of the main transcription factors in the comparison of trichomes vs. leaves. The significantly expressed transcripts (log_2_FoldChange > 1.0) of *MYB* (**A**), *NAC* (**B**), *WRKY* (**C**), *C2H2* (**D**), *homeobox-leucine zipper protein* (**E**), *bHLH* (**F**), *TCP* (**G**) and *WD40* (**H**) were identified, and their expression levels in the trichomes and tea leaves are represented by heat maps. The transcripts that were not detected with FPKM values are shown in white.

TFs, such as MYB, NAC, WRKY, C2H2, bHLH, and homeobox-leucine zipper protein, play pivotal roles in the regulation of secondary metabolism and trichome development. 2369 TFs were identified and classified into 66 types to further analyze the differential regulation between trichomes and leaves (Figure S2). A total of 117 MYB DEGs were screened, and 95 of those genes were differentially expressed in the trichomes vs. leaves comparison. Among these genes, 74 DEGs were significantly upregulated, and 21 DEGs were notably downregulated in trichomes (Figure 4A). 

We found that several *MYB* DEGs, such as TEA003067, TEA031473, TEA024116, TEA007100, TEA014321, TEA017098, TEA023817, TEA018834, TEA001697, TEA011677, TEA025707, TEA023311, and TEA026096, were not expressed in tea leaves, and TEA027578 was not transcribed in trichomes. A total of 108 *NAC* DEGs were identified, and 33 of those genes were significantly expressed between trichomes and leaves. Thirty of 33 DEGs were upregulated in trichomes, and the DEGs TEA013629, TEA013108, TEA022743, TEA007908, TEA015555, TEA013548, and TEA011210 were specifically expressed in trichomes (Figure 4B). Additionally, 40 *WRKY* DEGs were identified as being significantly differentially expressed between trichomes and leaves, including 33 upregulated and six downregulated DEGs in the trichomes vs. leaves dataset. The DEGs TEA027312, TEA012365, TEA027198, and TEA003077 were specifically expressed in trichomes, whereas TEA002329 was not detectable in trichomes (Figure 4C). Thirty-one *C2H2* zinc finger protein DEGs, which include 19 upregulated and 12 downregulated DEGs, were identified to be significantly expressed in the trichomes vs. leaves comparison (Figure 4D). Similarly, 16 of 24 significantly changed homeobox-leucine zipper protein DEGs were upregulated in the trichomes, and the rest of the DEGs were downregulated in the trichomes (Figure 4E). On the other hand, 33 *bHLH* DEGs were identified as being significantly differentially expressed between trichomes and leaves, 19 of 33 DEGs were downregulated, and the rest of the genes were upregulated in trichomes, and TEA011884 was not transcribed in trichomes (Figure 4F). Thirteen *TCP* DEGs, including six upregulated and seven downregulated DEGs, were significantly expressed in the trichomes vs. leaves comparison (Figure 4G). Moreover, 10 *WD40* DEGs, including seven upregulated and three downregulated DEGs, were also identified in the trichomes vs. leaves comparison (Figure 4H).

### 3.7. Other Key DEGs Identified from Tea Trichomes and Leaves

**Figure 5:**
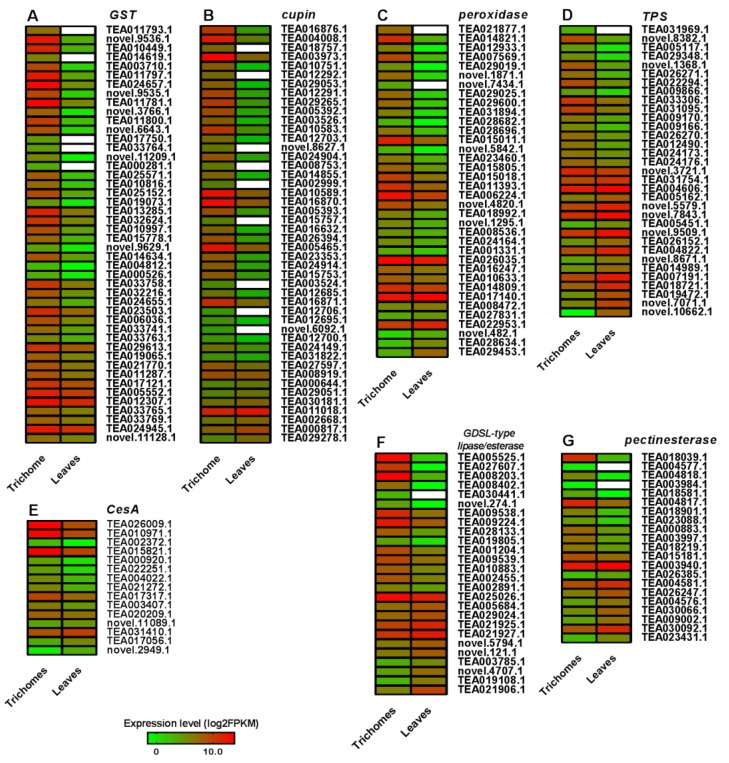
Expression patterns of other key DEGs in the comparison of trichomes vs. leaves. The significantly expressed transcripts (log_2_FoldChange > 1.0) of *glutathione S-transferase* (*GST*, **A**), *cupin superfamily proteins* (*cupin*, **B**), *pectinesterase* (**C**), *terpene synthase* (*TPS*, **D**), *cellulose synthase* (*CesA*, **E**), *GDSL-type lipase/esterase* (**F**), and *peroxidase* (**G**) were identified, and their expression levels in the trichomes and tea leaves are represented by heat maps. The transcripts that were not detected with FPKM values are shown in white.

The most changed DEGs in the gene families of *cupin superfamily proteins*, *GDSL-type lipase/esterase*, *glutathione S-transferase* (*GST*), *cellulose synthase* (*CesA*), *terpene synthase* (*TPS*), *peroxidase*, and *pectinesterase* were screened to further investigate other key DEGs between trichomes and leaves, and most DEGs in these families exhibited upregulated patterns (Figure 5).

Interestingly, all 46 significant *GST* DEGs showed upregulated patterns in the comparison of trichomes and leaves; in particular, the DEGs TEA003710, TEA026775, TEA011793, novel.3766, TEA014619, TEA017750, TEA000281, novel.5581, TEA019073, novel.11209, and TEA000526 were not detected in leaves (Figure 5A). In addition, the 46 *cupin* family genes were identified, 39 of which were significantly upregulated in the trichomes, and the DEGs TEA018757, TEA012292, novel.8627, TEA008753, TEA002999, TEA015757, TEA003524, TEA012706, and novel.6092 were not detected in leaves (Figure 5B). Interestingly, we found that 36 DEGs of *peroxidase* were differentially expressed, and 29 of those DEGs were upregulated in trichomes; particularly, the expression of TEA021877, TEA014821, TEA012933, TEA007569, TEA029019, novel.1871, and novel.7434 was high in the trichomes (Figure 5C). Seventeen upregulated and 16 downregulated *TPS* genes were screened from the trichome vs. leaves dataset. The DEGs of TEA031969 and novel.8382 showed trichome-specific expression patterns, whereas novel.10662 was not detected in trichomes (Figure 5D). Fifteen *CesA* genes were identified, including 11 upregulated and four downregulated DEGs in trichomes as compared with leaves (Figure 5E). Moreover, 26 DEGs belonging to *GDSL-type lipase/esterase* were identified, and all of them showed significantly upregulated expression patterns in trichomes (Figure 5F). Additionally, 21 DEGs of *pectinesterase*, including 12 upregulated and nine downregulated DEGs, were identified, and TEA004577 and TEA003984 were not transcribed in leaves, showing that they were trichome specific (Figure 5G).

### 3.8. Validation of Differential Expression Data

**Figure 6:**
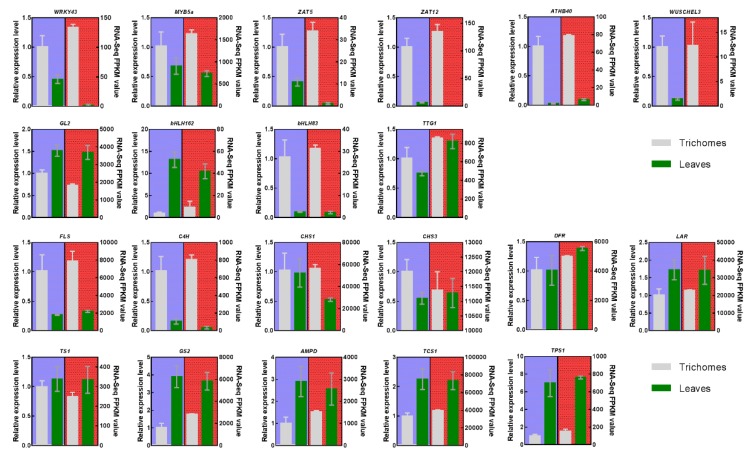
Quantitative RT-PCR validation. Twenty one genes of regulatory TFs and structural genes involved in the trichome development and major secondary metabolic pathways including *WRKY43*, *MYB5a*, *ZAT5*, *ZAT12*, *ATHB40*, *WUSCHEL3*, *GL2*, *bHLH162*, *bHLH83*, *TTG1*, *FLS*, *C4H*, *CHS1*, *CHS3*, *DFR*, *LAR*, *TS1*, *GS2*, *AMPD*, *TCS1*, and *TPS1* were selected for RT-qPCR determination. The *GAPDH* gene was used as an internal control.

21 DEGs regulatory TFs and structural genes that were involved in the trichome development regulation and major secondary metabolic pathways were selected and examined using RT-qPCR to validate the reliability of the RNA-Seq results. The RT-qPCR results exhibited similar expression patterns between trichomes and leaves as the RNA-Seq data, which suggested that our transcriptomic data are reliable and valid (Figure 6).

Moreover, we observed that the genes of *TCS1* and *TPS1* were highly expressed in leaves and related to secondary metabolite synthesis. The TFs *ZAT12*, *ATHB40,* and *WUSCHEL3* were specifically expressed in trichomes and they might be associated with trichome development in tea plants.

## 4. Discussion

It has been well established that trichomes (or hairs) are vital characteristics of tea germplasm that are important for tea quality formation in terms of tea appearance and tea infusion. Therefore, the metabolites in tea trichomes, which have been isolated from white tea, green tea, black tea, and yellow tea, have been broadly determined, and the trichomes have been found to contain certain levels of metabolites, such as catechins, amino acids, and caffeine [42]. Moreover, several studies have shown that trichomes also contain aromatic components [43]. However, we found that the trichomes that were used in previous studies were isolated from tea products that have encountered different manufacturing processes, which might impact the accuracy of the results.

In this study, we developed a method to isolate trichomes from fresh tea tissues for the first time to gain a more precise understanding of the metabolites in trichomes. We found that, as compared with tea leaves, the major metabolites of tea, including amino acids, catechins, and caffeine, had low contents in trichomes. In particular, the catechin content in leaves was over 50 times higher than that in trichomes, and the caffeine content in leaves were more than 30-fold higher than that in trichomes (Table 2); although the amino acids did not exhibit large fold changes between trichomes and leaves, the content of amino acids was 10 times higher in leaves than in trichomes (Table 1), which suggested that the metabolites were mainly synthesized in the mesophyll cells of leaf tissues. Trichomes are developed from epidermal cells. Previously, we showed that the tea trichomes were single-celled and non-glandular [7]. It has been well established that glandular trichomes are the major tissues that are involved in the synthesis and accumulation of secondary metabolites, such as in *Artemisia annua* and tomato [30,44], whereas non-glandular trichomes have scarcely any ability to synthesize and accumulate secondary metabolites. Hence, we could conclude that tea trichomes had slight or no ability to synthesize the key secondary metabolites of tea, in plants as catechins, caffeine, and amino acids (theanine). Recently, Li, et al. [45] reported that tea trichomes collected in September were rich in free catechins, caffeine, and amino acids, implying that the synthesis and accumulation of metabolites in tea trichomes might be affected by seasonal and cultivar differences.

RNA-Seq technology has been widely used to investigate the differential transcription of genes in glandular trichomes, such as those of tomato [33], *Salvia pomifera* [46], and *Artemisia annua* [26], which have been found to play multiple functions in plants. However, the non-glandular trichomes from other plants remain to be examined using this method, except for studies in *Arabidopsis* [13,15]. Hence, in this study, we compared the differential gene expression between trichomes and leaves in tea plants while using RNA-Seq technology for the first time. A total of 6560 DEGs, including 4471 upregulated and 2089 downregulated DEGs, were identified from the comparison of trichomes and leaves (Figure S1). Interestingly, in addition to there being more upregulated DEGs than downregulated DEGs, the fold change values of upregulated DEGs were also larger than those of the downregulated DEGs (Figure S1). In addition, these upregulated DEGs were significantly enriched in the “phenylpropanoid biosynthesis (ath00940)”, “glutathione metabolism (ath00480)”, “cutin, suberine and wax biosynthesis (ath00073)”, and “cysteine and methionine metabolism (ath00270)” pathways (Figure 2A), and these pathways play fundamental roles in trichome growth by supporting cytoskeleton formation and maintaining the structure of the cell wall. On the other hand, we found that the downregulated DEGs were significantly enriched in MF items, such as terpene synthase activity (GO:0010333), carbon-oxygen lyase activity, acting on phosphates (GO:0016838) (Figure 1B), and in the pathways of “photosynthesis (ath00195)” and “plant hormone signal transduction enrichment (ath04075)” (Figure 2B), indicating that the elementary functions of trichomes and leaves are different. While considering the structure of tea trichomes, it is impossible that trichomes have photosynthetic ability, and, therefore, they might not be able to synthesize metabolites, including amino acids, catechins, and caffeine.

Therefore, we proposed that the structural genes that are involved in the amino acid, catechin, and caffeine synthesis pathways are repressed or not transcribed in trichomes, thus causing the different contents of amino acids, catechins, and caffeine between trichomes and leaves (Table 1 and Table 2). Consistently, among 32 DEGs that were involved in the caffeine biosynthesis pathway, the expression levels of caffeine synthase (TEA010054) and theobromine synthase (TEA028049) were significantly downregulated in trichomes, whereas the rest of the DEGs had no significant changes between trichomes and leaves (Figure 3A). Similarly, in the theanine biosynthesis pathway, two *GS* DEGs (TEA032125, TEA028194) were notably repressed, and one *ADC* DEG (TEA032991) was induced in trichomes (Figure 3B). The *TS* DEGs (TEA015198) were also downregulated, but not significantly downregulated, in trichomes. *GS* shares high homology with TS, and both of them have the ability to synthesize theanine [47,48,49], reflecting, to some extent, that the content of theanine in trichomes was lower than that in leaves. In the catechin biosynthesis pathway, 41 of 78 DEGs were significantly upregulated, and only seven DEGs were markedly downregulated in the trichomes (Figure 3C). Although the structural genes that were involved in theanine, catechins, and caffeine pathways were detected in the trichomes, the key genes generally exhibited downregulated patterns that might influence the biosynthesis of these metabolites. On the other hand, several upregulated genes, such as cinnamate 4-hydroxylase (TEA016772, TEA014864) and phenylalanine ammonia-lyase (TEA003374, TEA014056, TEA003137, TEA014166, and TEA034008) (Figure 3C), which are critical for the phenylpropanoid pathway, participate in a variety of secondary metabolite biosynthesis pathways, especially lignin synthesis, which might be important for trichome growth [50,51].

It has been demonstrated that the phenylpropanoid and flavonoid biosynthesis pathways are regulated by different TF classes, such as MYB, WRKY, NAC, and bHLH, through modulating the expression of the related structural genes. Among these TFs, MYB-bHLH-WD40 repeat (MBW) ternary complexes have been illustrated to be one of the most important regulatory components that are involved in phenylpropanoid and flavonoid biosynthesis [52,53,54,55,56]. To date, certain MBW component genes that regulate flavonoid biosynthesis have been identified in tea plants. For instance, Sun, et al. [57] showed that R2R3-MYB *CsAN1* could interact with bHLH TFs (*CsGL3* and *CsEGL3*) and *CsTTG1* (a WD-repeat protein) to form an MBW complex to regulate anthocyanin accumulation; Liu, et al. [58] found that *CsWD40* (homologous to AtTTG1 TF) partners with bHLH (*CsGL3* and *CsTT8*) and MYB (*CsAN2* and *CsMYB5e*) TFs to form WBM complexes to control anthocyanin and proanthocyanidin biosynthesis; finally, the overexpression of certain tea plant MYB genes, such as *CsMYB5a*, *CsMYB5b*, *CsMYB5e*, *CsMYB5-1,* and *CsMYB5-2*, could alter anthocyanin and proanthocyanidin accumulation in other plants [59,60,61]. We found that most of the DEGs that were involved in the *MYB*, *NAC*, and *WRKY* classes were significantly upregulated in trichomes since tea trichomes usually exhibit glossy characteristics and might not have the capability to synthesize and store flavonoids and phenylpropanoids, especially anthocyanins and proanthocyanidins (Figure 4). The WBM complexes not only regulate flavonoid synthesis, but also modulate trichome development through different TFs competing to form WBM complexes [6,16,62]. Recently, Liu, et al. [58] indicated that the tea plant CsWD40 interacted with bHLH and MYB TFs to form WBM complexes that could alter anthocyanin accumulation and regulate trichome development. Nonetheless, known key TFs, such as *GL1*, *GL3*, *EGL3*, and *TTG1*, which were involved in trichome development, were not determined to be expressed at a significant level, although TFs, such as *TTG1* (TEA000080), were upregulated in trichomes. Consistently, Jakoby, et al. [15] found that the known key genes, such as *GL1* and *TTG2*, which are involved in trichome development, had low or no expression in mature trichomes in *Arabidopsis*. The reason for this result might be related to the spatiotemporal specificity of gene expression. For example, genes, such as *GL3*, are highly expressed in initiating trichome cells, but become less detectable or nonexistent in mature trichomes [63], indicating that these genes primarily function in trichome differentiation rather than in trichome growth. Unlike in other plants, trichome initiation is probably combined with tea bud development at an early stage, and the trichomes on the first tea leaf are lengthier than those on the bud. Therefore, these key regulatory genes are slightly or not differentially expressed in the comparisons of trichomes vs. leaves and hairy vs. hairless cultivars [7]. On the other hand, we found that several genes that are involved in the negative regulation of trichome development were downregulated in trichomes, such as *MYB82* (TEA023420), whose homologous gene was overexpressed in *Arabidopsis*, leading to reduced trichome numbers [10]. In this study, we found that some *MYB* (13), *NAC* (7), and *WRKY* (4) DEGs were trichome specific (Figure 4), and most trichome-specific genes were associated with cell wall synthesis, such as TEA013629 (homologues to *AtNST1* gene) [64,65] and TEA007243 (homologues to *AtMYB86* gene) [66], indicating that these TFs might be mainly involved in trichome development and growth regulation through modulating the cell wall or fiber synthesis. These trichome-specific TFs appear to participate in trichome elongation rather than initiation, and their potential functions in cell expansion and cell wall synthesis deserve further investigation.

In this study, we found that most gene family members, such as cupin superfamily proteins, *GDSL-type lipase/esterase*, *GST*, *CesA*, *TPS*, *peroxidase*, and *pectinesterase*, which are generally involved in cell wall synthesis and abiotic stress response, were significantly upregulated in trichomes (Figure 5). Similarly, previous transcriptome analysis also showed that the genes that were involved in these pathways were expressed at high levels in *Arabidopsis* trichomes [13,15]. Genes, such as *GST*, *CesA*, and *peroxidase*, are regarded as critical for cellulose and lignin synthesis, which are important for trichome cell proliferation [67,68,69], and trichomes are rich in cellulose and contain lignin [32]. Consistently, *CCR* and *CAD* were both markedly upregulated in the trichomes (Figure 3C) and have been reported to play a critical role in lignin biosynthesis [50,51]. In particular, these types of genes, including *cupin* superfamily proteins, *GDSL-type lipase/esterase*, and *peroxidase*, also have multiple functions in the plant stress response and cell wall synthesis [70,71,72,73,74], which might be correlated to the roles of trichomes in tea plants. Trichomes are thought to be the first physical barrier for protecting plants against insect and pathogen attack, lessening the heat load of leaves, reducing transpiration rates, and enhancing drought and cold resistance [75,76,77]. Generally, tea plants with high-density or long-length trichomes have a high ability to resist cold stress, drought stress, and biotic stress, including feeding by *Acaphylla theae* and leafhoppers [78]. Tea plants originate from warm areas in Southwest China, where natural conditions, including temperature and humidity, are suitable for plant growth, and they have been planted in more than 60 countries with a variety of adverse conditions, such as cold and drought [79,80,81,82]. It has been observed that the density and length of trichomes on wild-type tea plants were partially lower than on the cultivated species [83]. Currently, more than 300 tea plant cultivars have been selected in China, and none of them have glabrous tender shoots [84], which indicates that tea plant trichomes may evolve to adapt to environmental changes and mainly result from artificial selection.

Generally, tea products processed using tender hairy shoots are commonly recognized to have the “háoxiang” aroma, a term used to describe a fragrance that has been previously reported to might be secreted by tea trichomes. In this study, we detected the aromatic compounds in trichomes and observed that 26 out of 34 aromatic compounds, especially those regulating tea aromatic quality formation, such as linalool and its oxides, nerol, jasmine lactone, indole, methyl salicylate, and alpha-farnesene, were accumulated in significantly higher levels in tea leaves than in trichomes (Table 3). Interestingly, the contents of (*E*)-2-hexenol, hexyl butanoate, and trans-2-hexenyl hexanoate in trichomes were considerably higher than those in tea leaves. The contents of major terpene compounds, such as linalool, trans-nerolidol, and nerol, were low in trichomes (Table 3) given that nearly half of the *TPS* genes (16/33) were downregulated, and some of the genes were markedly upregulated in trichomes (Figure 5D), indicating that these upregulated *TPS* genes might be involved in other types of non-volatile terpenoid synthesis and that their functions are worthy of further investigation. Although the trichomes also had a high content of certain aromatic compounds, such as phytol, acetate, 3,5-dimethylbenzaldehyde, benzyl alcohol, and 2-hexenal, their contribution to the tea aroma might not be greater than that from the leaves. Specifically, we showed that tea trichomes do not appear to be capable of synthesizing secondary metabolites, and even if they could synthesize these compounds or other special compounds, the contents of these compounds in trichomes are very low when compared to those in leaves. Moreover, the tea manufacturing processes might not influence aromatic compound synthesis and secretion in tea trichomes since there are not enough substrates stored in the trichomes, and, therefore, the “háoxiang” aroma, which is regarded as a key feature of tender shoots, might not be released from trichomes but is closely related to trichomes. Hence, the function of trichomes in tea products seems to need reappraisal.

## 5. Conclusions

In this study, trichomes were first isolated from fresh tea plant shoots while using a novel method, and the difference between trichomes and tea leaves per se was uncovered from metabolite and gene expression patterns using RNA-Seq analysis. Our results indicate that tea trichomes might not have the ability to synthetize metabolites; when compared with tea leaves, the contents of the main catechins, amino acids, caffeine, and volatile aromatic compounds in trichomes were low. Importantly, a total of 6560 DEGs, including 4471 upregulated and 2089 downregulated DEGs, were identified from the comparison of trichomes and leaves, and the molecular regulatory differences between trichomes and leaves were explored. In addition, certain tea trichome-specific genes, especially different types of TFs, were identified, showing that tea trichomes are closely related to the environmental adaptation of tea plants. The findings from the present study can provide fundamental information for understanding the role of trichomes in tea quality formation and in tea plant growth and development.

## Figures and Tables

**Table 1:** Contents of amino acids in trichomes and processed leaves (mg/g dry weight).

Amino Acids	Trichomes	Processed Leaves	Quantitative Analysis
γ-aminobutyric acid	0.0049 ± 0.00024	0.052 ± 0.003 **	Std.
Serine	0.030 ± 0.0012	n.d.	Std.
Proline	0.002 ± 0.0001	0.041 ± 0.0024 **	Std.
Valine	0.0011 ± 0.00007	0.014 ± 0.0014 **	Std.
Threoine	n.d.	0.047 ± 0.0072	Std.
Leucine	0.00085 ± 0.00006	0.014 ± 0.0008 **	Std.
Isoleucine	0.00094 + 0.00016	0.013 ± 0.00048 **	Std.
Asparagine	0.01 ± 0.0015	0.117 ± 0.008 **	Std.
Aspartic acid	0.052 ± 0.011	0.77 ± 0.008 **	Std.
Glutamine	0.035 + 0.017	0.79 ± 0.045 **	Std.
Lysine	0.054 ± 0.004	0.25 ± 0.01 **	Std.
Glutamic acid	0.045 ± 0.003	3.9 ± 0.24 **	Std.
Methionine	0.00025 + 0.00003	0.0077 ±0.0002 **	Std.
Histidine	0.0077 + 0.001	0.031 ± 0.006 **	Std.
Phenylalanine	0.00024 ± 0.00001	0.021 ±0.0002 **	Std.
Arginine	0.011 + 0.0019	0.138 ± 0.01 **	Std.
Tyrosine	0.00014 ± 0.00001	0.01 ± 0.0015 **	Std.
Tryptophan	0.00037 ± 0.00001	0.087 ± 0.002 **	Std.
Theanine	0.76 ± 0.015	4.49 ± 0.16 **	Std.
Total	1.011 ± 0.18	10.56 ± 1.35 **	Std.

Values shown are means ± SD (*n* = 3); n.d., non-detectable; ** indicates significant difference at the 0.01 level; Std., quantitative analysis based on authentic standard

**Table 2:** Contents of the main catechins, caffeine, and rutin in trichomes and processed leaves (mg/g dry weight).

Compounds	Trichomes	Processed Leaves	Quantitative Analysis
Catechin gallate	0.0013 ± 0.0002	0.080 ± 0.004 **	Std.
Epicatechin	0.037 ± 0.0024	4.2 ± 0.2 **	Std.
Gallocatechin gallate	0.0082 ± 0.001	0.086 ± 0.094 **	Std.
Epigallocatechin	0.085 ± 0.014	17.75 ± 0.88 **	Std.
Epicatechin gallate	0.54 ± 0.055	28.82 ± 2.02 **	Std.
Epigallocatechin gallate	1.1 ± 0.15	82.7 ± 4.03 **	Std.
Caffeine	1.008 ± 0.056	31.23 ± 1.83 **	Std.
Rutin	0.058 ± 0.0028	0.39 ± 0.033 **	Std.

Values shown are means ± SD (*n* = 3) ** indicates significant difference at the 0.01 level; Std., quantitative analysis based on authentic standard.

**Table 3:** Contents of the main aroma compounds in fresh trichomes and processed leaves (nmol/g fresh weight).

Compounds	Retention Time (min)	Processed Leaves	Trichomes	Quantitative Analysis
2-Hexenal	8.775	128.048 ± 29.649 **	12.495 ± 0.389	Std.
beta-Ocimene	9.79	2.571 ± 0.058 **	0.471 ± 0.020	Std.
Cyclohexanone	10.915	0.428 ± 0.036	0.546 ± 0.054	Internal Std.
2-Heptanol	11.695	0.018 ± 0.002 **	0.006 ± 0.000	Internal Std.
1-Hexanol	12.75	3.193 ± 0.213 **	1.067 ± 0.122	Std.
(Z)-3-Hexenol	13.765	52.281 ± 10.676 **	7.377 ± 0.224	Std.
(*E*)-2-Hexenol	14.495	0.035 ±0.003	0.235 ± 0.017 **	Internal Std.
Hexyl butanoate	15.075	0.006 ± 0.000 **	0.003 ± 0.000	Internal Std.
trans-Linalool oxide (furanoid)	15.8	8.936 ± 0.152 **	0.747 ± 0.036	Std.
cis-3-Hexenyl butyrate	16.515	0.009 ± 0.001	0.012 ± 0.002	Internal Std.
trans-Linalool oxide (furanoid)	16.74	0.806 ± 0.097 **	0.071 ± 0.009	Internal Std.
trans-2-Hexenyl Butyrate	16.975	0.088 ± 0.002 **	0.009 ± 0.001	Internal Std.
2-Ethylhexanol	17.29	1.650 ± 0.287	2.059 ± 0.148	Internal Std.
Benzaldehyde	18.43	2.375 ± 0.082	2.284 ± 0.149	Std.
Linalol	19.2	65.021 ± 11.499 **	7.089 ± 0.102	Std.
Hexyl hexanoate	21.36	0.014 ± 0.005	0.023 ± 0.003 **	Internal Std.
Benzeneacetaldehyde	22.17	2.380 ± 0.071	2.533 ± 0.107	Std.
(*Z*)-3-Hexenyl hexanoate	22.735	0.016 ± 0.001 **	0.008 ± 0.000	Internal Std.
trans-2-Hexenyl hexanoate	23.155	0.005 ± 0.002	0.009 ± 0.000 **	Internal Std.
L-alpha-Terpineol	23.835	0.009 ± 0.000 **	0.005 ± 0.000	Internal Std.
alpha-Farnesene	25.545	1.907 ± 0.041 **	0.234 ± 0.047	Std.
Linalool oxide (pyranoid)	25.7	0.263 ± 0.019 **	0.126 ± 0.012	Internal Std.
Methyl salicylate	26.2	36.350 ± 3.010 **	2.442 ± 0.164	Std.
3,5-Dimethylbenzaldehyde	27.325	27.510 ± 1.245 **	22.307 ± 0.531	Std.
Nerol	28.115	99.662 ± 5.727 **	5.439 ± 0.356	Std.
Benzyl alcohol	28.85	53.378 ± 5.342 **	13.318 ± 0.102	Std.
2-Phenylethanol	29.835	99.699 ± 8.944 **	17.958 ± 0.199	Std.
Benzyl nitrile	30.345	0.021 ± 0.002 **	0.010 ± 0.000	Internal Std.
Phytol, acetate	30.675	316.534 ± 36.643 **	44.950 ± 0.881	Std.
Jasmone	30.895	0.019 ± 0.001 **	0.003 ± 0.000	Internal Std.
3,7,11,15-Tetramethyl-2-hexadecen-1-ol	31.475	0.059 ± 0.006 **	0.022 ± 0.001	Internal Std.
trans-Nerolidol	33.43	3.503 ± 0.165 **	0.679 ± 0.045	Std.
Jasmine lactone	38.855	0.714 ± 0.010 **	0.441 ± 0.039	Std.
Indole	42.94	10.371 ± 0.176 **	3.896 ± 0.113	Std.

Values shown are means ± SD (*n* = 3); ** indicates significant difference at the 0.01 level; Std., quantitative analysis based on authentic standard. Internal Std., quantitative analysis based on ethyl *n*-decanoate (internal standard).

## Supplementary Materials

The following are available online at https://www.mdpi.com/2218-273X/10/2/311/s1, Figure S1: Global distribution of DEGs identified from the comparison of trichomes vs. leaves; Figure S2: Transcription factors identified from RNA-Seq analysis; Table S1: Primers used for qRT-PCR validation; Table S2: Summary of the RNA-Seq data obtained from trichomes and leaves; Table S3: Expression levels of key genes involved in the caffeine biosynthesis pathway, theanine biosynthesis pathway, and catechin biosynthesis pathway.
