# Supplementary material for: Integrative Transcriptomic and Metabolic Analyses Provide Insights into the Role of Trichomes in Tea Plant (Camellia Sinensis)
Source: Biomolecules. 2020 Feb 16;10(2):311. doi: 10.3390/biom10020311 (PMC7072466; doi:10.3390/biom10020311)
Supplement: Supplementary file 1 [file biomolecules-10-00311-s001.zip › supplementary files/Supplementary table S1.docx]

**Table S1** Primers used for qRT-PCR validation

| Gene_id | Gene name | Forward primer | Reverse primer |
| --- | --- | --- | --- |
| TEA013771.1 | *CsWRKY43* | GACATTGACTGGGTCAGCCT | ACTCCTAGTCTGGAACGCGA |
| TEA004608.1 | *CsMYB5a* | AACCTCAGCCAGAAATGCAA | TGGAGTTCAAAGTTATGACAGCC |
| TEA012015.1 | *CsZAT5* | TTCTTGTGACACCGCCATCA | CCGGAGGAAAACTCCGATCC |
| TEA020477.1 | *CsZAT12* | TGGCCAACTGCTTGATGCTA | TCAACCGCAACCTCTTGTGA |
| TEA013535.1 | *CsATHB40* | GGTTTGGATCCACGCCAAGT | TTCCTTGAGCGTCAACACCT |
| TEA012714.1 | *CsWUSCHEL3* | AGGAAGCTCAGTAGGCAACTC | TTCGCACCTTTCTGCTGGAT |
| TEA022963.1 | *CsGL2* | TGCAGTCACTGTCCAAACGA | CCGAAGGCAATACGGCAATG |
| TEA013874.1 | *CsbHLH162* | GAGACGATCCGTGTGCTTCA | CGAAGCTCCTCCACATTCCA |
| TEA029404.1 | *CsbHLH83* | AGCAAATGCAAATGCGACCA | TGAGGGTCCTTGGATGGAGT |
| TEA016601.1 | *CsFLS* | GCTAGGTGGGGATGAGTTGG | GAGGGCATTGGGGATGTACT |
| TEA016772.1 | *CsC4H* | CATCCAACCGTTCAACGCAA | GGTGTGTGAAGCCTCAAGGT |
| TEA007191.1 | *CsTPS1* | GATTTGTTTCCAGGCTCTTGATGA | GTTTTGGCAAGTGCCCTGAG |
| TEA015791.1 | *CsTCS1* | CCTCTTCAAAGGCCTGTCGT | ATGCCAAGCCTTCTCTGCTT |
| TEA017069.1 | *CsAMPD* | TCCACTTGACAAAGGAGCCC | TCCCAATCCAGTGCGACTTC |
| TEA023340.1 | *CsCHS1* | GAGGCACGTGCTAAGTGAGT | CCTGGTCCGAACCCGAATAG |
| TEA023333.1 | *CsCHS3* | CCGTGAAGTGGGCCTTACAT | ACCAGGGTGTGCAATCCAAA |
| TEA000080.1 | *CsTTG1* | AAACTCCGTGACCTACGACTCCCCG | CTTGGTGGGAGGGTAAGGGTGTTCG |
| TEA028194.1 | *CsGS2* | GTCGCTAATCGCGGTTGTTC | AGCCAATAAGGCCGTCACAA |
| TEA015198.1 | *CsTS1* | GTTGATGTTTCTGGGCAGCA | CTCACCCACACCAGTCAGAT |
| TEA032730.1 | *CsDFR* | TGAGGGTTGCTCTGGAGTGTTT | CGTTGATTGTCGGCTTGATTAC |
| TEA027582.1 | *CsLAR* | GAAAATTCACCATCAAAACCGT | TCACTGCTGCTGCTGCTAGTAG |
