# Supplementary material for: Integrative Transcriptomic and Metabolic Analyses Provide Insights into the Role of Trichomes in Tea Plant (Camellia Sinensis)
Source: Biomolecules. 2020 Feb 16;10(2):311. doi: 10.3390/biom10020311 (PMC7072466; doi:10.3390/biom10020311)
Supplement: Supplementary file 1 [file biomolecules-10-00311-s001.zip › supplementary files/Supplementary table S2.docx]

**Table S2** Summary of the RNA-Seq data obtained from trichomes and leaves

| Samples | Total raw reads (Mb) | Total clean reads (Mb) | Total clean bases (Gb) | Error rate（%） | Clean Reads Q20 (%) | Clean Reads Q30 (%) | Total mapping ratio (%) | Unique mapping ratio (%) |
| --- | --- | --- | --- | --- | --- | --- | --- | --- |
| Leaves_1 | 51.44 | 50.18 | 7.53 | 0.03 | 97.9 | 93.96 | 90.50% | 85.23 |
| Leaves_2 | 59.124 | 57.3 | 8.60 | 0.02 | 98.05 | 94.33 | 90.39 | 85.09 |
| Leaves_3 | 70.304 | 68.79 | 10.32 | 0.03 | 97.92 | 93.99 | 90.13 | 85.00 |
| Trichome_1 | 62.134 | 60.82 | 9.12 | 0.03 | 97.85 | 93.92 | 86.58 | 81.43 |
| Trichome_2 | 61.474 | 59.98 | 9.00 | 0.03 | 97.75 | 93.66 | 86.85 | 81.6 |
| Trichome_3 | 57.084 | 55.97 | 8.39 | 0.03 | 97.86 | 93.82 | 86.55 | 81.4 |
