# Supplementary figures and images for: Integrative Transcriptomic and Metabolic Analyses Provide Insights into the Role of Trichomes in Tea Plant (Camellia Sinensis)
Source: Biomolecules. 2020 Feb 16;10(2):311. doi: 10.3390/biom10020311 (PMC7072466; doi:10.3390/biom10020311)

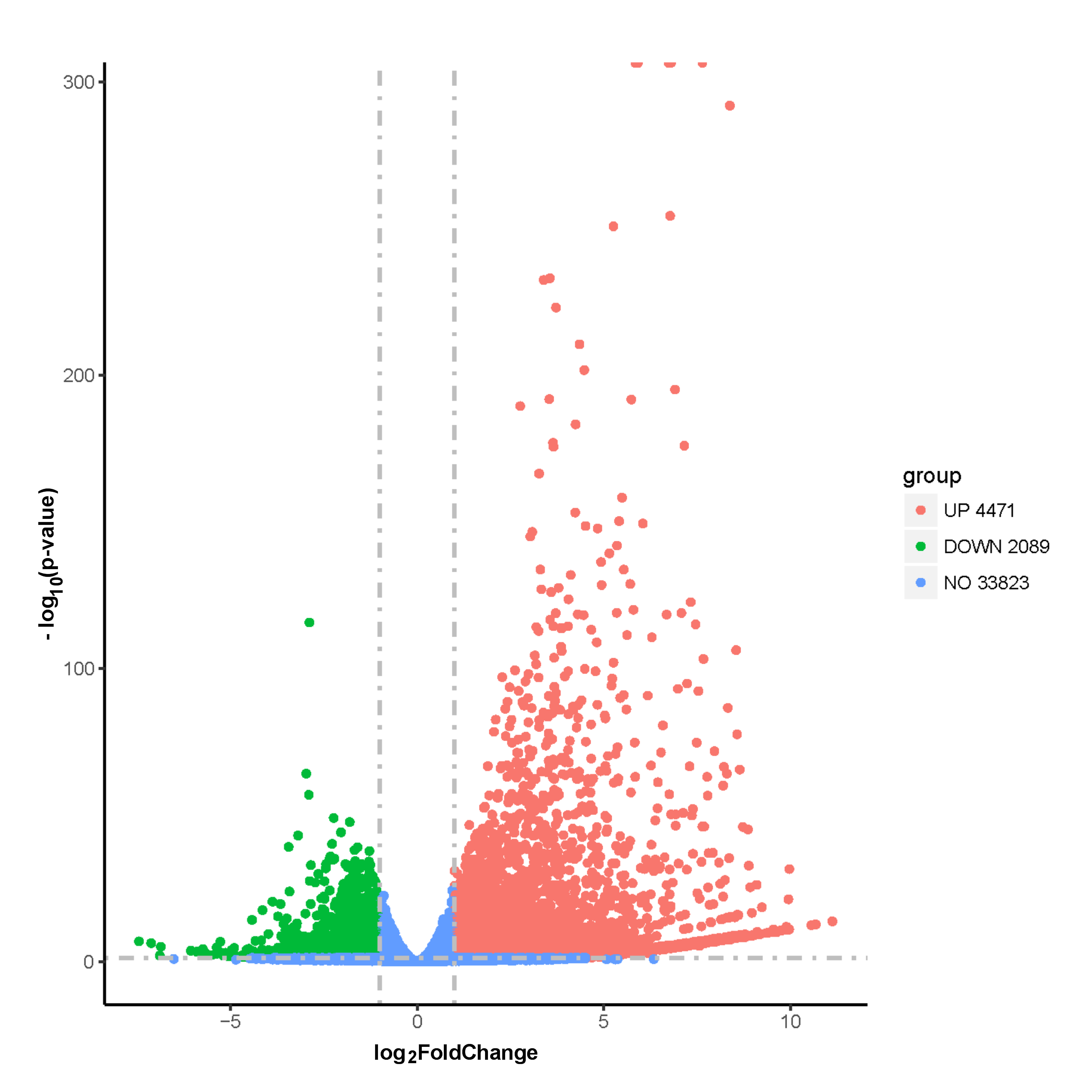

Supplement: Supplementary file 1 [file biomolecules-10-00311-s001.zip › supplementary files/Supplementary figure S1.tif]

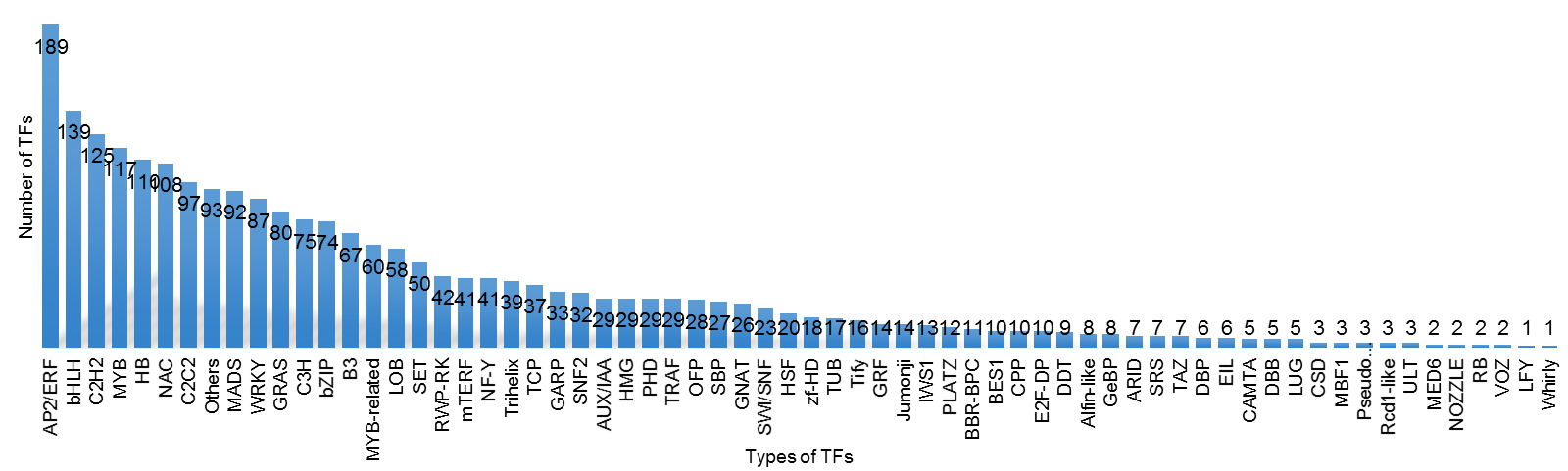

Supplement: Supplementary file 1 [file biomolecules-10-00311-s001.zip › supplementary files/Supplementary figure S2.tif]
